# Alkylation of Complex Glycine Precursor (CGP) as a Prebiotic Route to 20 Proteinogenic Amino Acids Synthesis

**DOI:** 10.3390/molecules29184403

**Published:** 2024-09-16

**Authors:** Chiaki Kuroda, Kensei Kobayashi

**Affiliations:** 1 Department of Chemistry, Rikkyo University, Nishi-Ikebukuro, Toshima-ku, Tokyo 171-8501, Japan; 2 Department of Chemistry and Life Science, Graduate School of Engineering Science, Yokohama National University, Tokiwadai, Hodogaya-ku, Yokohama 240-8501, Japan; kobayashi-kensei-wv@ynu.ac.jp; 3 School of Science, Tokyo Institute of Technology, 2-12-1 Ookayama, Meguro-ku, Tokyo 152-8550, Japan

**Keywords:** prebiotic synthesis, early earth, complex glycine precursor, polyglycine, proteinogenic amino acids

## Abstract

It is not known why the number of proteinogenic amino acids is limited to 20. Since Miller’s experiment, many studies have shown that amino acids could have been generated under prebiotic conditions. However, the amino acid compositions obtained from simulated experiments and exogenous origins are different from those of life. We hypothesized that some simple precursor compounds generated by high-energy reactions were selectively combined by organic reactions to afford a limited number of amino acids. To this direction, we propose two scenarios. One is the reaction of HCN with each side-chain precursor (the aminomalononitrile scenario), and the other is alkylation of the “complex glycine precursor”, which is the main product of proton irradiation of the primordial atmosphere (the new polyglycine scenario). Here, selective formation of the 20 amino acids is described focusing on the latter scenario. The structural features of proteinogenic amino acids can be described systematically. The scenario consists of three stages: a high-energy reaction stage (Gly, Ala, Asn, and Asp were established); an alkylation stage (Gln, Glu, Ser, Thr, Val, Ile, Leu, and Pro were generated in considerable amounts); and a peptide formation stage (Phe, Tyr, Trp, His, Lys, Arg, Cys, and Met were selected due to their structural advantages). This scenario is a part of the evolution of Garakuta World, in which many prebiotic materials are contained.

## 1. Introduction

It is well known that the number of proteinogenic amino acids in life is limited to 20: alanine (Ala), arginine (Arg), asparagine (Asn), aspartic acid (Asp), cysteine (Cys), glutamic acid (Glu), glutamine (Gln), glycine (Gly), histidine (His), isoleucine (Ile), leucine (Leu), lysine (Lys), methionine (Met), phenylalanine (Phe), proline (Pro), serine (Ser), threonine (Thr), tryptophan (Trp), tyrosine (Tyr), and valine (Val) (alphabetical order). However, it is not known why these were selected.

Presumably, the 20 were determined before the birth of the last universal common ancestor (LUCA). In 1953, Miller reported that amino acids were produced by spark discharges in a gas mixture of CH_4_, NH_3_, H_2_, and H_2_O [1]. Here, three of the proteinogenic amino acids (Gly, Ala, and Asp), together with two non-proteinogenic ones (β-alanine and α-aminobutyric acid (αABA)), have been obtained. Since this report, many studies have been conducted to show that amino acids could be abiotically formed. The energies that were used to synthesize bioorganic compounds were spark discharges simulating lightning [2,3,4], ultraviolet light [5], heat from volcanoes [6], radioactivity from the Earth crust [7], shock waves by meteorite impacts [8,9], galactic cosmic rays [10], and solar energetic particles (SEPs) [11]. Starting materials used ranged from strongly reducing gas mixtures (e.g., a mixture of CH_4_, NH_3_, and H_2_O) [1] to non-reducing gas mixtures (e.g., a mixture of CO_2_, N_2_, and H_2_O) [9]. It was shown that reducing gas mixtures generally yielded more amino acids than neutral gas mixtures [12], but slightly reducing gas mixtures could yield various amino acids if we considered such energies as SEPs [11].

In addition to the endogenous production of amino acids, exogenous delivery of amino acids has been considered as starting materials for the first life. Kvenvolden et al. reported that Murchison meteorite (CM2 carbonaceous chondrite) contained indigenous amino acids, including such proteinogenic amino acids as Gly, Ala, Val, Pro, and Glu, together with some non-proteinogenic amino acids [13]. Experiments simulating interstellar ice analogs yielded amino acids [14,15,16,17]. Recently, various amino acids were detected in returned samples from the asteroid Ryugu [18], and it was suggested that some organic compounds were formed in the interior of asteroids. Experiments simulating such environments also yielded amino acids [19].

In these days, analytical techniques are improved, and a large number of organic compounds have been identified in the products of experiments simulating prebiotic conditions and extracts from meteorites. Glavin et al. summarized the list of amino acids found in meteorites and in the products of simulation experiments [20]. Now, it is recognized that 12 proteinogenic amino acids out of 20 have been detected in them: Gly (C_2_), Ala, Ser (C_3_), Asp, Thr (C_4_), Val, Glu, Pro (C_5_), Leu, Ile (C_6_), Phe, and Tyr (C_9_). In addition to them, more than 80 non-proteinogenic amino acids have been detected in meteorites: All of the 5 isomers of αABA and all of the 12 isomers of Val have been identified [21]. The rule that an exponential decline in amount with the increasing carbon number was found [22], which means the concentration of such proteinogenic amino acids as C_5_, C_6_ and C_9_ was far lower than that of simpler (C_2_ and C_3_) amino acids.

**Figure 1:**
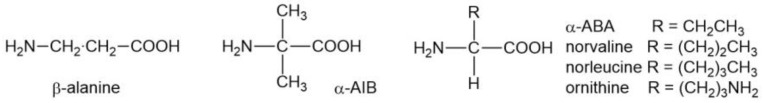
Some non-proteinogenic amino acids.

As shown above, the amino acid compositions obtained from simulated experiments are different from that of life. Various non-proteinogenic amino acids, such as β-alanine, αABA, α-aminoisobutyric acid (αAIB), and norvaline, have often been obtained (Figure 1). Although several reactions, including Strecker reaction, have been proposed as prebiotic pathways to amino acids, it is difficult to explain the generation of the 20 amino acids systematically [23,24,25]. Attempts to explain the selection of the 20 based on simulated experimental products have been made [26,27], but the mystery remains unsolved.

Amino acids having functional groups such as His and Tyr are essential for functionalized proteins. These amino acids are often found in active centers of various enzymes. However, their origin is still unclear, as most of the proteinogenic amino acids found in experimental products and meteorite extracts have hydrocarbon side chains. In 1955, Akahori proposed that peptides consisting of various amino acids were formed by the alkylation of polyglycine [28], but the detailed alkylation process for each amino acid was not described there.

**Scheme 1:**
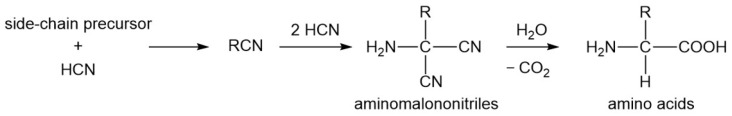
Formation of amino acids from HCN and the side-chain precursor (the aminomalononitrile scenario).

We hypothesized that proteinogenic amino acids were generated by organic reactions in early Earth. Because many organic reactions are selective for products, we thought that some simple precursor compounds generated by high-energy reactions were selectively combined to afford a limited number of amino acids. Recently, one of us proposed a plausible route to the 20 amino acids from hydrogen cyanide (HCN) and each side-chain precursor via alkylated aminomalononitriles (the aminomalononitrile scenario) (Scheme 1) [29].

Alkylation of a complex amino acid precursor, generated by proton irradiation of the early Earth atmosphere [25], is an alternative possible route to the 20 amino acids (the new polyglycine scenario). The difference between the two scenarios is the different structural origins of the amino acid body, but the side-chain precursors are mostly the same. Because the details of the new polyglycine scenario have not been described previously, here, we focus on it, which better explains the generation of functionalized amino acids. The difference in the two scenarios will be discussed briefly.

## 2. Basic Scheme of the New Polyglycine Scenario

**Scheme 2:**
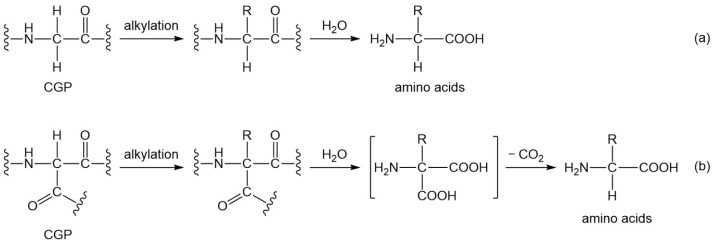
Formation of amino acids by the alkylation of complex glycine precursor (CGP) (the new polyglycine scenario). (**a**,**b**) are the reaction from the simplest structure and malonic acid derivatives, respectively.

When gas mixtures simulating the early Earth atmosphere were irradiated with high-energy protons (as a model of solar energetic particles from the young sun), amino acid precursors were formed, which, after hydrolysis, yielded glycine as the major amino acid product [11]. When interstellar ice analogs (frozen mixtures of methanol, ammonia, and water) were treated with high-energy heavy ions to simulate possible reactions in extraterrestrial environments, macromolecular amino acid precursors were formed, which again yielded mainly glycine after hydrolysis [30]. It was suggested that the high-molecular-weight organics that were formed in these experiments were mostly “complex glycine precursors” (hereafter abbreviated as CGP). Therefore, alkylation of CGP followed by hydrolysis is a plausible entry to the 20 proteinogenic amino acids. Probably, CGP is composed of various complex structures. Among them, the simplest structure is shown in Scheme 2a. Another plausible structure is a malonic acid derivative (Scheme 2b). From the latter structure, amino acids are generated after decarboxylation.

The side-chain precursors for the 20 amino acids are listed in Table 1. These are almost the same as those in the aminomalononitrile scenario. Most of the reactions of CGP with the side-chain precursors may be too slow to proceed under typical laboratory reaction conditions. However, because CGP is stable (more stable than amino acids [25]), the alkylation reactions conceivably proceed slowly without decomposition.

Some structural features of the proteinogenic amino acids are listed in Table 2. If these structures can be explained systematically, we believe that the scenario is the origin of the proteinogenic amino acids. Explanation of features vii and viii is particularly important, because, when amino acids were produced directly from simple inorganic substances, the smaller compounds would be more. In fact, amino acids in meteorites are exponentially depleted with the increasing carbon number among the congeners [20].

Features i and ii in Table 2 are evident from Scheme 2. In Scheme 2b, since the process involves decarboxylation, the α-carbon always has a hydrogen atom. In Scheme 2a, feature ii can be explained by steric hindrance in the second alkylation and peptide formation reactions (see Section 5 for the latter). In the next section, the generation of each amino acid is described in detail, including the reasons for the structural features.

## 3. Formation of the 20 Proteinogenic Amino Acids

### 3.1. Four Simple Amino Acids (Gly, Ala, Asn, and Asp)

Gly is the hydrolysis product of CGP and has been experimentally proven [11,25]. The formation from HCN is also a possible route [31]. Ala has been obtained as the major product under specific conditions [32]. Asp and Asn are the substitution products of CGP with Gly and glycinamide, respectively (the formal reaction is 2 Gly → Asp + NH_3_). It is likely that both Asp and Asn were produced in significant amounts, because Gly is the major product of high-energy reactions.

### 3.2. Three Aliphatic Amino Acids (Val, Ile, and Leu)

**Scheme 3:**
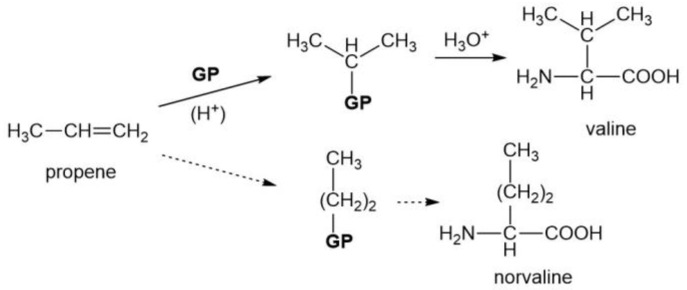
A plausible prebiotic route to valine from propene. GP = glycine precursor.

**Figure 2:**
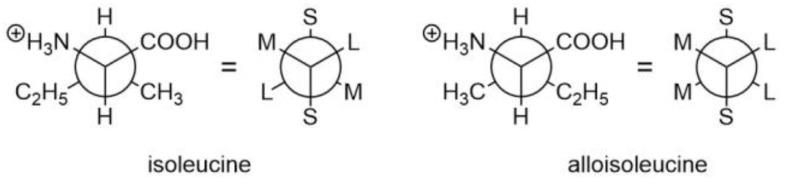
Conformation of isoleucine and alloisoleucine.

Val and Ile are the products of addition reactions to propene and butene (1-butene, (*E*)- and (*Z*)-2-butene), respectively. Markovnikov’s rule explains the absence of amino acids with unbranched hydrocarbon chains (norvaline and norleucine; Figure 1) (feature v). Scheme 3 shows the pathway by which Val, rather than norvaline, is produced from propene (GP = glycine precursor). The preferential formation of Ile over its diastereomer, alloisoleucine, is the result of steric hindrance (Figure 2). The absence of αABA can be explained by the fact that its precursor, ethylene, is less reactive than alkylated alkenes (feature iv). Leu is an addition product to methylpropene (=isobutene), but the reaction is anti-Markovnikov. This is because a Markovnikov addition did not occur due to steric hindrance. The presence of structurally similar amino acids (Val, Leu, and Ile) originates from different precursors (feature xiii). The alkenes appeared here are products of high-energy reactions. Some simple hydrocarbons have been obtained from inorganic gases in simulated experiments [15].

### 3.3. Four Oxygenated Amino Acids (Gln, Glu, Ser, and Thr)

**Scheme 4:**
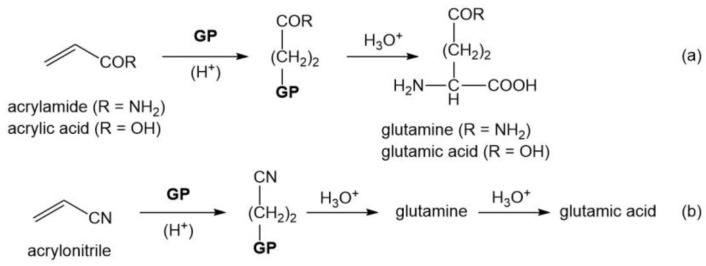
A plausible prebiotic route to glutamine and glutamic acid from acrylic compounds. (**a**) and (**b**) are the reaction from acrylamide/acrylic acid and acrylonitrile, respectively.

The conjugate addition of CGP to acrylamide or acrylic acid affords Gln or Glu, respectively (Scheme 4a) (feature ix). The addition of CGP to acrylonitrile followed by hydrolysis is an alternative route (Scheme 4b). Both acrylamide and acrylic acid are hydrolysis products of acrylonitrile, which is an addition product of acetylene with HCN. The addition of HCN to acetaldehyde followed by dehydration is another route to acrylonitrile. The difference between Asp and Glu (Asn and Gln) is only one methylene, but these are incorporated independently (feature xiii).

**Scheme 5:**
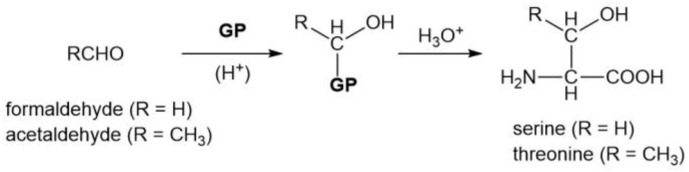
A plausible prebiotic route to serine and threonine from simple aldehydes.

The side-chain precursors of Ser and Thr are formaldehyde and acetaldehyde, respectively (Scheme 5). Ser has often been obtained in various simulated experiments [23]. Acetaldehyde is the precursor of both Thr and Gln/Glu and is a product from CH_4_ and CO [33]. The preferential formation of Thr over its diastereomer can be explained by hydrogen bonding.

### 3.4. Four Aromatic Amino Acids (Phe, Tyr, His, and Trp)

**Scheme 6:**
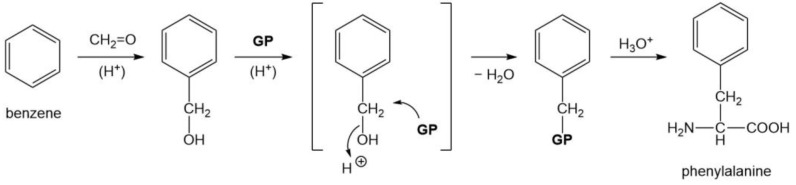
A plausible prebiotic route to phenylalanine from benzene.

The side-chain precursors of aromatic amino acids are benzylic compounds (Table 1). As an example, the formation of Phe is shown in Scheme 6. Benzylalcohol (**1**, X = OH) is the most probable precursor, which is a hydroxymethylation product of benzene. Nucleophilic substitution reaction at the benzylic position is much easier than that on the aromatic ring. This is the reason why all four aromatic amino acids have one methylene and why amino acids with an aromatic ring directly attached to the α-carbon are absent (feature vi).

The formation of Tyr starts from phenol. Because OH group is an *ortho*-*para*-directing group, and because the *ortho*-position is hindered, the hydroxymethylation of phenol mainly occurs on the *para*-position to afford **2** (X = OH). This is the reason why Tyr is a *para*-substituted compound (feature x). Similarly, His and Trp are products from imidazole and indole via benzylic compounds **3** and **4**, respectively. Imidazole is known to be generated from gaseous molecules easily [25]. Trp is the largest amino acid in molecular size and has rarely been detected in the product mixtures of various simulated experiments [23]. One of the reasons for the incorporation of Trp may be that hydroxymethylation of indole is much easier than that of benzene. The substitution reaction of indole occurs dominantly at the C-3 position (feature xi).

As can be seen from Table 1, the precursors of aromatic amino acids are structurally more complex than simple alkenes, and presumably, they were less abundant in early Earth. We think that the peptide formation stage played an important role in the selection of these amino acids as the proteinogenic members (see Section 4).

### 3.5. One Secondary Amino Acid (Pro)

**Scheme 7:**
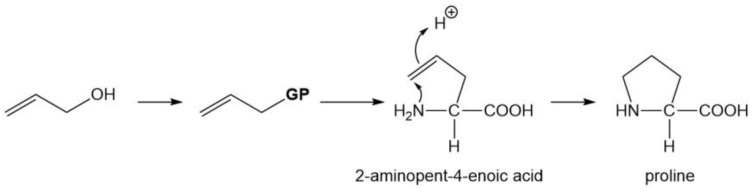
A plausible prebiotic route to proline from allylalcohol.

The side-chain precursor of Pro is an allylic compound, such as allylalcohol. The reaction of CGP with the precursor compound yields 2-aminopent-4-enoic acid after hydrolysis (Scheme 7). Then, the amino group adds to the terminal double bond to afford Pro (feature xii). This means that aromatic amino acids and Pro are produced by the same mechanism.

### 3.6. Two More Basic Amino Acids (Lys and Arg)

**Scheme 8:**
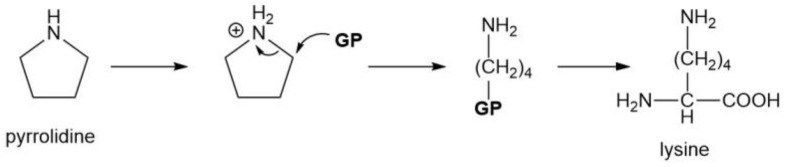
A plausible prebiotic route to lysine from pyrrolidine.

The side-chain precursor of Lys is pyrrolidine, the smallest stable cyclic amine (Scheme 8). Similarly, compound **5**, the side chain precursor of Arg, is the smallest cyclic guanidine. The use of cyclic compounds as precursors is the reason why Lys and Arg have four and three methylene groups, respectively (features vii and viii). This route rationalizes why ornithine (Figure 1) is not a member.

In contrast to Lys and Arg, amino acids generated from related acyclic aliphatic precursors are not proteinogenic members. This is because aliphatic substitution is less facile than benzylic or allylic substitution, and the amino acids generated by this type of reaction were not abundant. For more reasons for the incorporation of Lys and Arg, see Section 4.

### 3.7. Two Sulfur-Containing Amino Acids (Cys and Met)

The side chain precursor of Cys is thioformaldehyde, and its reaction is analogous to the formation of Ser. The origin of Met is unclear. See Section 4 for the incorporation of these sulfur-containing amino acids.

### 3.8. Why Are There No Other Precursor Compounds

It is difficult to answer this question. Carbonyl is a reactive functional group. However, only formaldehyde and acetaldehyde are the amino acid precursors (Table 1). Probably, the reaction of CGP with acetone, for example, hardly occurred due to steric hindrance. For aromatic compounds, aniline, for example, is much less reactive in the hydroxymethylation step under acidic conditions.

## 4. Abiotic Peptide Formation

**Scheme 9:**
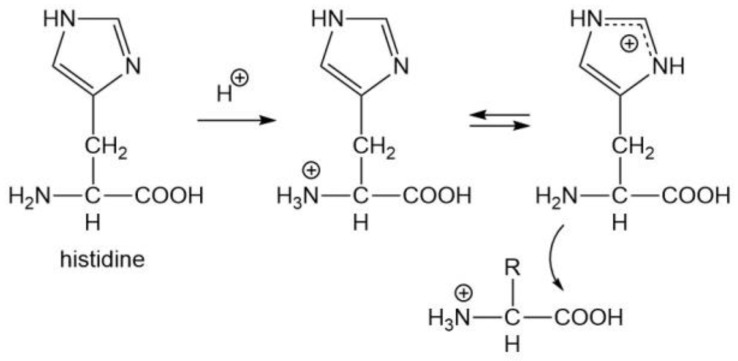
A plausible abiotic peptide formation of histidine under acidic conditions.

As shown in Table 1, the precursors of some amino acids are either relatively complex (Phe, Tyr, His, Trp, and Arg); less reactive (Lys); or rare molecules (Cys and Met). Probably, these amino acids were not abundant in early Earth and were incorporated in the peptide formation stage due to their structural advantages, namely, hydrophobic effect (Phe, Tyr, and Trp); basic side chains (His, Lys, and Arg); and sulfur-containing side chains (Cys and Met). In basic amino acids, H^+^ can be transferred from the amino group to the side chain. As a result, the lone pair on the amino nitrogen revives, making it available to attack the carboxy group of another amino acid. As an example, the reaction of His is shown in Scheme 9.

Cys can be a member by forming peptides by a reaction like “native chemical ligation” [34]. Namely, the thiol group can attack another amino acid (or peptide), even though the amino group is ionized under acidic conditions.

**Scheme 10:**
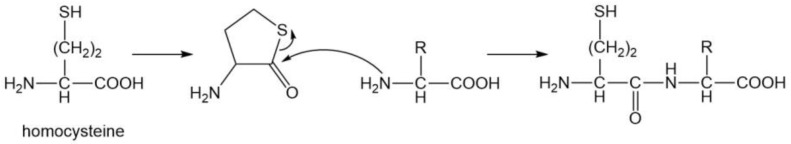
A plausible abiotic peptide formation of homocysteine via its thiolactone.

We speculate that Met was originally homocysteine (Hcy). Hcy forms thiolactone and easily reacts with another amino acid (Scheme 10). This is the first step of peptide formation. We do not think it is a coincidence that the Met-encoding codon and the start codon are identical in modern organisms. Probably, Hcy was a minor amino acid in early Earth. However, once generated, Hcy can survive through peptide formation. The generation of Met may have occurred after the establishment of the basic biological system, including RNA–amino acid correlation. If so, it is difficult to speculate on the chemical evolution route to Met.

## 5. Asymmetry

**Figure 3:**
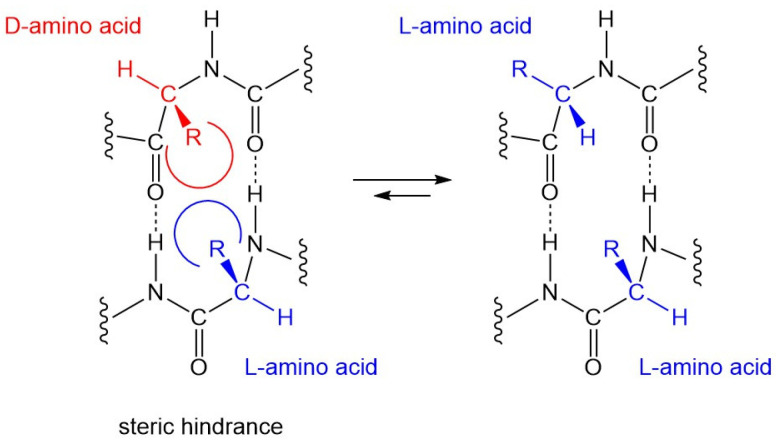
Isomerization in the α-helix.

All proteinogenic amino acids except for Gly are l-form (feature iii). Although several mechanisms for the origin of homochirality have been documented [20], the mystery remains unclear. From the perspective of organic chemistry, here, two possible processes are shown. One is isomerization in the α-helix. As shown in Figure 3, α-helices composed of both d- and l-amino acids have steric hindrance somewhere in the helix. This problem is eliminated in helices composed of only one enantiomer, either d or l. The steric hindrance in α-helices also explains the absence of α, α-disubstituted amino acids, which cause steric hindrance with both d- and l-amino acids (feature ii).

**Figure 4:**
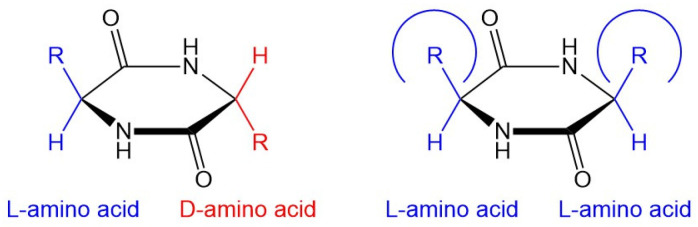
Diketopiperadines.

Another candidate to explain asymmetry in amino acids is through the formation of diketopiperazines. Dipeptides composed of d- and l-amino acids easily form diketopiperazines without a steric problem (Figure 4). Because diketopiperazines are stable, further peptide formation reactions hardly occur. In contrast, dipeptides composed of the same asymmetry form diketopiperazines less easily due to steric hindrance and react with other amino acids to form tripeptides. Steric hindrance is important for bulky aromatic amino acids, especially Trp.

It was reported that some amino acids in meteorites were l-dominant, suggesting that the l-amino acid world might have originated from an excess of l-amino acids from space [20,23,35]. However, the process that led to the l-amino acid world remains unclear.

## 6. Reaction Conditions and Environments

The new polyglycine scenario explains the formation of functionalized amino acids more favorably than the aminomalononitrile scenario, because their side-chain precursors are less volatile or water-soluble. All of the above alkylation reactions proceed under acidic conditions. Thus, supposing that the early ocean was acidic, all the reactions could be explained uniformly. For the formation of aliphatic amino acids (Val, Ile, and Leu), the aminomalononitrile scenario is likely, because all reactants (alkenes and HCN) are gas molecules.

The peptide formation step could also proceed under acidic conditions. Peptide formation is a dehydration reaction, which hardly occurs in aqueous solutions. However, even in water, dehydration coupling proceeds efficiently in a micellar system [36]. Supposing the presence of amphiphilic (or surfactant) molecules in the early ocean, the peptide formation reaction might have proceeded smoothly in a hydrophobic environment. However, its detailed reaction system is not clear. Peptide formation from alkylated CGPs is another possible route, but the reaction is not a simple dehydration, because each amino acid unit is bonded to other units. A hydrophobic interaction between amino acids and amphiphilic molecules is an important factor in the selection of hydrophobic amino acids as proteinogenic members.

Many organic molecules were present in early Earth, constituting the “Garakuta World” [25]. There is no doubt that not only proteinogenic but also many non-proteinogenic amino acids were contained in the Garakuta World. We believe that these diverse amino acids gradually concentrated into those that were abundant or easily formed peptides. Then, the world of the 20 proteinogenic amino acids in life was established over a long period of time. The story described here is a part of evolution of the Garakuta World.

In the present life, there are some minor amino acids other than the 20, although they are only found in certain organisms. Presumably, these non-proteinogenic amino acids are products generated at a later stage. Furthermore, the current biosynthetic routes are different from those described above. These suggest the presence of further evolution steps after the birth of life from the Garakuta World.

## 7. Conclusions

The formation of the 20 proteinogenic amino acids can be explained systematically. Not only the presence of the 20 amino acids but also the absence of some related amino acids is rationalized. All the reactions described here can be found in many organic chemistry textbooks. The reactions consist of three stages. The first is high-energy reactions by which CGP and various side-chain precursors are generated. Four small amino acids, Gly, Ala, Asn, and Asp, were mostly established at this stage. The second is the alkylation of CGP. Eight of the twenty proteinogenic amino acids (Gln, Glu, Ser, Thr, Val, Ile, Leu, and Pro) were produced in considerable amounts. Then, amino acids that are advantageous for peptide formation (Phe, Tyr, Trp, His, Lys, Arg, Cys, and Met) were selected from a pool of minor amino acids.

The new polyglycine scenario has a problem in that the detailed structure of the main body part (CGP) has not yet been identified. Furthermore, the production pathways of most of the precursors (Table 1) are unknown. Now, we described only amino acids, but actual life is composed of various materials, including sugars, bases, amphiphilic compounds, and so on. The interactions among various prebiotic life molecules are an important factor to be solved. We look forward to future research.

## Figures and Tables

**Table 1:** Side-chain precursors for proteinogenic amino acids ^1^.

Amino Acid	Side-Chain Precursor
Asn	H_2_NCH_2_CONH_2_
Asp	H_2_NCH_2_COOH (Gly)
Val	CH_2_=CHMe
Ile	CHMe=CHMe, CH_2_=CHEt
Leu	CH_2_=CMe_2_
Gln	CH_2_=CHCONH_2_, CH_2_=CHCN
Glu	CH_2_=CHCOOH, CH_2_=CHCN
Ser	HCHO
Thr	MeCHO
Phe	**1** ^2^
Tyr	**2** ^2^
His	**3** ^2^
Trp	**4** ^2^
Pro	CH_2_=CHCH_2_X ^2^
Lys	pyrrolidine
Arg	**5**
Cys	CH_2_=S
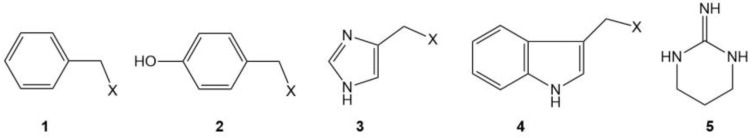	

^1^ The side-chain precursors of Gly, Ala, and Met are not listed. See text for these amino acids. ^2^ X = leaving group.

**Table 2:** Structural features of proteinogenic amino acids.

No.	Features
i	All are α-amino acids. β-Alanine, for example, is not a member.
ii	One hydrogen atom is attached to the α-carbon. αAIB, for example, is not a member.
iii	All, except for Gly, have l-configuration with respect to the α-carbon.
iv	As aliphatic side chains, methyl (Ala), propyl (Val), and butyl (Ile/Leu) are present, but ethyl (αABA) is absent.
v	Only branched alkyl groups are present for the propyl and butyl side chains. Norvaline and norleucine are not members.
vi	All aromatic amino acids have one methylene between the aromatic ring and the α-carbon.
vii	Lys has four methylenes. Its shorter analogues, such as ornithine, are not members.
viii	Arg has three methylenes. Its shorter analogues are not members.
ix	Gln and Glu have two methylenes.
x	Tyr has an OH group on the *para*-position. Its *meta*- and *ortho*-isomers are not members.
xi	Trp has an indole group substituted at C-3.
xii	One amino acid (Pro) has a secondary amino group.
xiii	Structurally similar amino acids are present (Asp/Gln, Asp/Glu, and Val/Ile/Leu).
